# Massage for simple obesity: A protocol for systematic review

**DOI:** 10.1097/MD.0000000000024336

**Published:** 2021-02-12

**Authors:** Yazheng Pang, Kai Wang, Shucheng Chen, Tian Huang, Mengsen Zhang, Bin Zhang, Juan Yu

**Affiliations:** a School of Acupuncture-Moxibustion and Massage, Shandong University of Traditional Chinese Medicine; b Shandong Provincial Third Hospital, Jinan; c School of Nursing, The Hong Kong Polytechnic University, Hong Kong; d Pediatric Tuina Health Care Clinic, Shandong University of Traditional Chinese Medicine Affiliated Hospital, Jinan, China.

**Keywords:** massage, simple obesity, systematic review, Traditional Chinese Medicine, tuina

## Abstract

**Background:**

Obesity has become the most serious public health problem in developed and developing countries, and simple obesity accounts for approximately 95% of the total cases. This study aims to assess the effects and safety of massage therapy for the treatment of simple obesity.

**Methods:**

We will search foreign and Chinese databases, including PubMed, EMBASE, MEDLINE, CENTRAL, CNKI, WanFang Data, CBM, and VIP from the inception of the coverage of these databases to July 2020. Cochrane's collaboration tool will be used to assess the quality of the studies. RevMan 5.3 software will be used for the data analysis.

**Results:**

This study will evaluate whether massage therapy is an effective intervention for simple obesity.

**Conclusion:**

This study will provide evidence regarding whether massage therapy is beneficial for treating simple obesity in humans.

**PROSPERO registration number:**

NO.CRD42020197635.

## 1 Introduction

Obesity is a chronic progressive health problem that affects approximately 107.7 million children and adolescents worldwide.^[1–5]^ At present, obesity has become the most serious public health problem in developed and developing countries. Obesity, AIDS, and drug and alcohol abuse have become four new social medical problems.^[6]^ Obesity is related to endocrine and metabolic diseases and is associated with multiple complications, such as type 2 diabetes, cardiovascular disease, hypertension, stroke, and various cancers.^[7]^

Obesity can be divided into simple obesity and secondary obesity, and simple obesity accounts for approximately 95% of the total. Simple obesity mainly refers to the condition in which the body's caloric intake is greater than caloric consumption, which causes excessive accumulation of fat in the body and results in excessive weight. More than 70% of people who develop obesity before puberty will still show obesity in adulthood.^[5,8,9,10]^ This finding shows how important it is to control weight and to prevent obesity overall.

Obesity is closely related to our health and it is necessary to seek effective preventive measures to reduce the occurrence of related diseases in the pediatric stage. As a traditional Chinese medicine treatment, acupuncture and massage are based on meridian theory, and massage therapy is aimed at stimulating specific acupoints or meridians on the surface of the body to achieve therapeutic effects similar to acupuncture.^[11]^ Massage is widely applied in clinical practice because it is economical, convenient, and safe. In the process of massage treatments, therapists touch the skin with their hands and penetrate pressure into the human body by pressing, rubbing, kneading, grasping, and pinching to achieve the purpose of treatment.^[12]^ From the TCM perspective, the pathogenesis of simple obesity is Spleen deficiency, which can be relieved by specific approaches of massage.

In recent years, there have been increasingly more studies reporting the application of massage on the treatment of simple obesity, and massage therapy may have beneficial effects on simple obesity, but there has been no systematic review specifically on massage therapy for simple obesity in adolescents and adults. Therefore, we decided to fill the gap in the literature to provide experts and patients with up-to-date evidence that can be used to make a rigorous evaluation of the effectiveness of this therapy and to guide clinical practice. We conducted this systematic review and meta-analysis to summarize the current evidence of the effects and safety of massage therapy for the treatment of simple obesity.

## 2 Methods

This review aims to evaluate the effects and safety of massage therapy for the treatment of simple obesity. Our protocol has been registered in PROSPERO register of systematic review network (No .CRD42020197635). All steps of this systematic review will be performed according to the Cochrane Handbook (5.2.0).

### 2.1 Data sources and retrieval strategy

To evaluate the efficacy of massage in the treatment of simple obesity, we will search foreign and Chinese databases, including PubMed, EMBASE, MEDLINE, CENTRAL, CNKI, WanFang Data, CBM, and VIP from inception of the coverage of these databases to July 2020. For English databases, the following group terms will be used for searching (massage therapy OR massage OR anmo OR acupressure OR tuina OR manipulate) AND (Simple obesity OR overweight OR fat). For Chinese databases, the equivalent Chinese group terms will be searched. We will not impose any language restrictions.

The databases will be searched by combining the subject words with random words. The retrieval strategy is shown in Table 1 using PubMed retrieval as an example. The search terms were adapted appropriately to conform to different syntax rules of different databases.

**Table 1:** Retrieval strategy of PubMed.

Number	Search Term
#1	“Massage therapy” [MeSH] OR “massage ” [Title/Abstract]OR “anmo” [Title/Abstract] OR “acupressure” [Title/Abstract] OR “tuina” [Title/Abstract] OR “manipulate” [Title/Abstract]
#2	“Simple obesity” [Title/Abstract] OR “overweight” [Title/Abstract] OR “fat” [Title/Abstract]
#3	“Randomized controlled trial” [Title/Abstract] OR “Controlled clinical trial “ [Title/Abstract]
#4	#1 AND #2 AND#3

### 2.2 Eligibility criteria

The PICOS principles were given full consideration to establish the inclusion and exclusion criteria of this systematic review.

#### 2.2.1 Types of participants

Participants who were diagnosed with simple obesity regardless of age, gender, and race. All appropriate definitions of overweight or obesity based on BMI and body weight exceeds the normal will be accepted.

#### 2.2.2 Types of interventions and comparators

The series of massage therapy involves many techniques, such as pressing, rubbing, pinching, kneading, pushing, arc-pushing, twisting, and shaking. Moreover, many distinctive complex massage manipulations are organically combined.^[13]^ Studies that combine massage with other therapies, such as acupuncture, moxibustion, drugs, and physical interventions will be included if they can prove that massage is effective.

#### 2.2.3 Types of outcomes

The primary outcomes include the effective rate of clinical symptoms, BMI, and BW reduction. The secondary outcomes will assess F%, WC, and HC.

#### 2.2.4 Types of studies

The selected articles should be about RCTs that compared massage with a control group to assess the efficacy of massage on simple obesity. We will include trials that assessed massage in comparison with control interventions, including inactive control (e.g., placebo, no treatment) and active controls (e.g., medication and acupuncture). Conference literature and dissertations, reviews, case series, case reports, experience summaries and animal research will be excluded.

### 2.3 Study selection and data extraction

**Figure 1:**
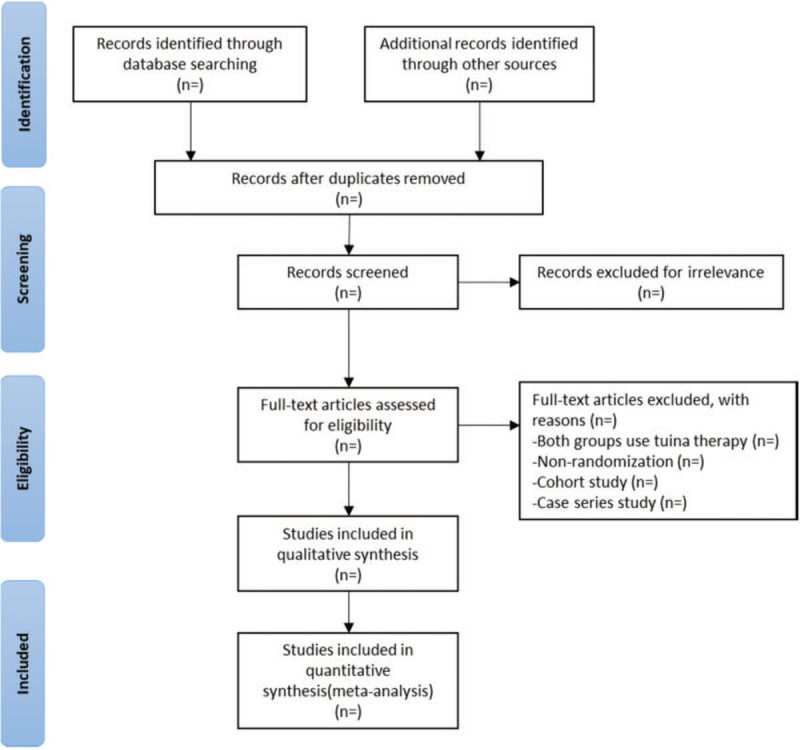
PRISMA flow chart.

Endnote X9.0 will be used to manage the retrieved studies. As shown in Figure 1, the study selection will be divided into 2 steps and will be completed by 2 researchers (Yazheng Pang and Kai Wang). Preliminary screening involves eliminating repeated and unqualified studies by reading the title and abstract. Rescreening involves reading through the full text and selecting the studies according to the inclusion and exclusion criteria. Disagreements between the two reviewers will be resolved by discussion and by consulting a third reviewer (Tian Huang) when necessary.

According to the Cochrane Handbook for Systematic Reviews of Interventions, the following information on the included studies will be extracted by two reviewers independently (Yazheng Pang and Tian Huang): general information, participant information, sample size, eligibility criteria, diagnostic system, intervention and controls, outcome measures and adverse events. When there are disagreements between the two reviewers in the process of data extraction, they can be resolved by discussion and by consulting the third reviewer when necessary.

### 2.4 Risk of bias assessment

Two researchers (Mengsen Zhang and Bin Zhang) will evaluate the quality of the included studies under the guidance of the Cochrane Collaboration tool (RevMan 5.3) for RCTs.^[14,15]^ The 2 reviewers will complete this work independently. If there is any disagreement, they can discuss and resolve it, and the third researcher can be involved when necessary. The Cochrane Collaboration tool assesses the following seven domains: generation and allocation of random sequences, blinding of participants and personnel, blinding of outcome assessor, incomplete outcome data, selective reporting and other risk biases. According to the above evaluation items contained in the literature, we will designate the “unclear risk of bias” or “low risk of bias” or “high risk of bias” to provide a better understanding of the included study quality.^[16]^

### 2.5 Statistical analysis

The statistical analysis will be conducted using Cochrane Collaboration's Review Manager Software (Review Manager 5.3). RevMan can perform a meta-analysis and present the results graphically.^[17]^ For dichotomous variables, we will report relative risks (RR) with 95% confidence intervals (CIs). For continuous variables, we will pool the data using the mean difference (MD) with a 95% CI for the same outcome measure, and the standardized mean difference (SMD) with a 95% CI if different rating scales will be used in different trials for the same outcome.

#### 2.5.1 Assessment of heterogeneity

Heterogeneity of trials will be assessed using Cochran's Q statistic with a p-value cut-off of 0.10.^[18]^ If the heterogeneity test result is *P* > .10, the fixed effect model can be used to calculate the combined statistics. If *P* ≤ .10, the random effect model will be used for data analysis to consider the heterogeneity among the trials.

#### 2.5.2 Analysis of subgroups

If the heterogeneity is high, we will also perform subgroup analysis to calculate the combined statistics.^[19]^ The following subgroup analyses will be considered: gender, age, intervention time, intervention cycle, and course of the disease.

#### 2.5.3 Sensitivity analysis

When sufficient data are available, sensitivity analysis will be performed to test the robustness of the primary outcomes, which includes assessing the quality of the methods, the quality of the studies, and the impact of sample size and missing data.

#### 2.5.4 Assessment of publication biases

We will use funnel plots to observe whether the system evaluation results are biased if 10 or more studies are included in meta-analysis. If the data are biased, the funnel plots will appear dissymmetric. A dissymmetric funnel plot indicates high risk of reporting bias, while a symmetric funnel plot indicates low risk.

#### 2.5.5 Confidence in cumulative evidence

The Grading of Recommendations Assessment, Development and Evaluation (GRADE) system will be used to assess the overall quality of the evidence derived from the included studies.^[20]^ In addition, the result will be divided into high, moderate, low and very low quality.

### 2.6 Ethics and dissemination plans

Given that there will be no patients recruited and no data gathered from patients, ethical approval is not required for the conduct of this review. The results of this research will be disseminated in a peer-review journal.

## 3 Discussion

Massage, a very ancient modality of treating diseases, has been practiced throughout the history of human civilization and plays an undeniable role in disease resistance. Massage stimulates the meridians and acupoints to regulate yin and yang, order descent and ascent, tonify the deficient and purge the excess, and generate warming and clearing effects to restore health.^[13]^ Massage is widely applied to simple obesity in modern times and increasingly more clinical trials have been published, but the available high-quality trials are still insufficient. We hope that the results of this study may provide evidence regarding the massage treatment of simple obesity.

## Author contributions

**Conceptualization:** Juan Yu, Yazheng Pang, and Kai Wang.

**Data curation:** Tian Huang, Mengsen Zhang, and Bin Zhang.

**Formal analysis:** Yazheng Pang and Kai Wang.

**Methodology:** Shucheng Chen and Juan Yu.

**Software:** Tian Huang and Kai Wang.

**Supervision:** Shucheng Chen and Juan Yu.

**Writing – original draft:** Yazheng Pang and Kai Wang.

**Writing – review & editing:** Juan Yu.

## Abbreviations

Abbreviations: BMI = body mass index, BW = body weight, CBM = Chinese Biological Medicine Database, CENTRAL = Cochrane central register of controlled trials, CIs = confidence intervals, CNKI = China National Knowledge Infrastructure, F% = body fat distribution rate, HC = hip circumference, MD = mean difference, RR = relative risks, SMD = standardized mean difference, VIP = Chinese Scientific Journal Database, WC = waist circumference.
